# The Shortage of Domestic Help: Work of National Importance

**Published:** 1918-05-04

**Authors:** Adeline E. Cable

**Affiliations:** Matron. The Infirmary, Salisbury


*To the Editor of* The Hospital.

### The Shortage of Domestic Help: Work of National Importance.

*To the Editor of* The Hospital.

Sir,—I am venturing to write and ask if by means of an urgent article in your influential paper, quoted perhaps in the leading daily papers, it would be possible to call attention to the great shortage of domestic help in the civil hospitals, and the disastrous effect it must have upon the nurses, unless something can be done to remedy it.

That the domestic problem is everywhere acute one knows, but surely the domestic work in our hospitals is somewhat apart, and calls for patriotic work on the part of capable maids.

Under present conditions valuable time much needed for nursing has to be given by the nurses to rough domestic work simply because no maids are obtainable.

If the urgency of the need could be emphasised, especially from the standpoint of the limitation lack of domestic help places on the greater comfort of our wounded, one feels some scheme might be devised which would attract suitable girls, or older women. It is surely work of national importance to conserve the strength of the nurses, and to make it possible for them to devote all their time to their own definite work.

Yours faithfully,

Adeline E. Cable, Matron.

The Infirmary, Salisbury, April 28, 1918.

